# Identification of Carotid Artery Microstructure and Plaque Rupture Using C-Arm Cone-Beam CT: A Case Report

**DOI:** 10.3389/fneur.2021.801683

**Published:** 2021-12-24

**Authors:** Jia Dong, Xuesong Bai, Adam A. Dmytriw, Lanlan Xuan, Tao Wang, Xia Lu, Yao Feng, Liqun Jiao

**Affiliations:** ^1^Department of Neurosurgery, Xuanwu Hospital, Capital Medical University, Beijing, China; ^2^Department of Interventional Neuroradiology, Xuanwu Hospital, Capital Medical University, Beijing, China; ^3^China International Neuroscience Institute (China-INI), Beijing, China; ^4^Neuroendovascular Program, Harvard Medical School, Massachusetts General Hospital, Boston, MA, United States; ^5^Department of Pathology, Anqing Hospital, Anhui Medical University, Anqing, China

**Keywords:** neovascularization, microcalcification, plaque rupture, ischemic stroke, C-arm cone-beam CT

## Abstract

C-arm cone-beam computed tomography (CBCT) offers a high imaging resolution with a wide range of contrast to visualize vessels, soft tissue, and bone. We report the usefulness of CBCT in observing neovascularization, microcalcification, and plaque rupture. A 56-year-old man presented with vertigo and complain of an unsteady gait for 5 months. Catheter angiography demonstrated right severe carotid stenosis with irregular filling defect, which on high-resolution MRI showed vessel wall enhancement. The CBCT showed high density structures and linear contrast enhancement from the vascular lumen to the plaque, related to microstructure and plaque rupture. Carotid endarterectomy was performed, and histopathology confirmed that the high-density areas represented neovascularization and microcalcification, with linear enhancement representing plaque rupture. This is the first report showing that microcalcifications and plaque rupture can be identified by CBCT. Thus, CBCT can be used as a promising supplement to current imaging modalities to evaluate plaque components more accurately.

## Introduction

Carotid artery atherosclerosis is a risk factor for ischemic stroke. The maximal stenosis of a vascular lumen has traditionally been used as the principal diagnostic criterion to recommend treatment. However, an increasing number of studies have found that plaque vulnerability is equally, more important in assessing the risk of stroke (1). Neovascularization and microcalcification, both indictors of vulnerable plaque, play an important role in the pathogenesis of plaque progression and rupture (2–5). Neovascularization is strongly associated with the vasa vasorum (VV), a specialized form of microvasculature mainly arising from the adventitia and traversing the intimal-medial layer of large arteries and veins (6). Hyperplasia of the adventitial VV occurs in the early phases of the atherosclerotic process, whereas in advanced stages of atherosclerosis, the appearance of new microvessels extends to the media and intima, constituting ectopic neovascularization (6). These newly formed vessels are usually immature, irregular, fragile, and prone to extravasation due to the compromised structural integrity (7, 8). The leaky vessels are associated with intraplaque hemorrhage and ulcerations, which further contribute to the instability of the plaque and thrombosis (7). At the same time, microcalcification is strongly related to macrophage infiltration and could correlate with the active stage of inflammation. Microcalcification on or within the fibrous cap of atherosclerotic plaque increases local mechanical stress and leads to an increased risk of plaque rupture and subsequent thrombosis (5). Thus, it may be propitious to identify the microstructure of atherosclerosis with advanced neuroimaging.

C-arm cone-beam computed tomography (CBCT) offers a high imaging resolution with a wide range of contrast to visualize vessels, soft tissue, and bone (9, 10). In this case, we demonstrated the potential usefulness of CBCT in observing neovascularization, microcalcification, and plaque rupture.

## Case Presentation

**Figure 1:**
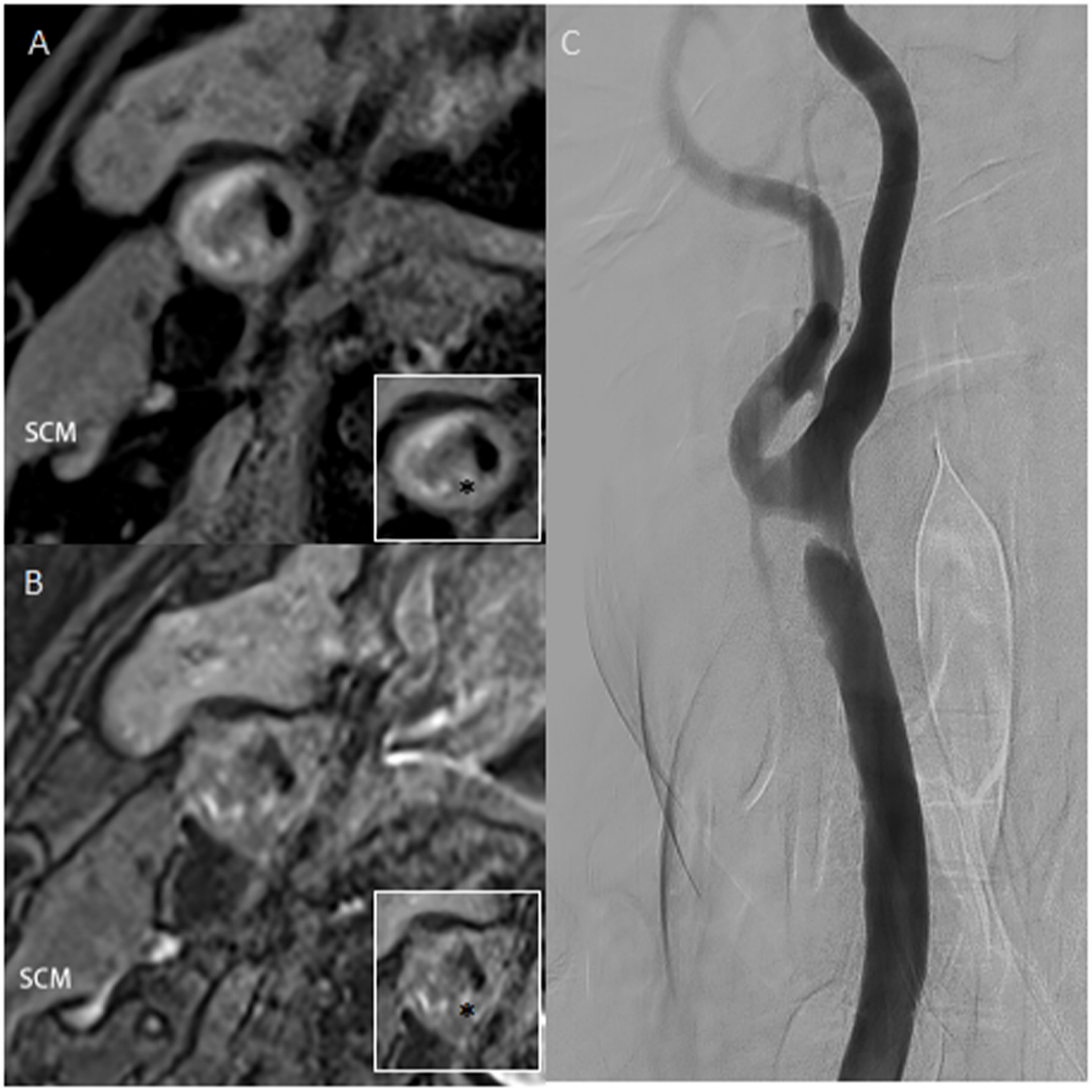
**(A,B)** High-resolution T1 and T2 weighted images of plaque. The ratio of the highest signal intensity of carotid plaque to sternocleidomastoid muscle was more than 2.0. **(C)** Digital subtraction angiography confirms an irregular filling defect. Asterisk indicates the plaque component. SCM, sternocleidomastoid muscle.

A 56-year-old man presented with vertigo and an unsteady gait for 5 months. Magnetic resonance imaging showed infarction in a bilateral cerebellar and occipital distribution. Subsequent digital subtraction angiography (DSA) confirmed left intracranial vertebral artery stenosis of 75% and right common carotid artery (CCA) stenosis of 80% with a luminal irregular filling defect (Figure 1). To further assess the morphology of the plaque, a decision was made to acquire CBCT, with secondary reconstruction over the region of the stenosis. CBCT was performed with the flat-detector angiography system (FD20/20; Philips Medical Systems). Based on motorized rotational angiography acquisition, the scanning time was 20 s, 30 projection images/s at 80 kV (dose index of 49 mGy), and the detector format used was 22 × 22 cm. In order to display plaque components, the carotid artery stenosis was positioned in the ISO center of the imaging field and the guiding catheter was placed at the proximal of the stenosis. The iodized contrast medium was diluted to 17% with heparin salt. The injection rate was 3. 0 ml/s for 23 s, with a 3 s delay to maximize contrast filling at the target lesion level. Multiplanar reconstruction of CBCT volume showed plaque microchannels with a high density of neovascularization and microcalcification. High-resolution MRI (HR-MRI) T1 and T2 sequences showed vessel wall enhancement suggesting vulnerable plaque. Taken together with images from HR-MRI, this was highly suggestive of unstable plaque.

**Figure 2:**
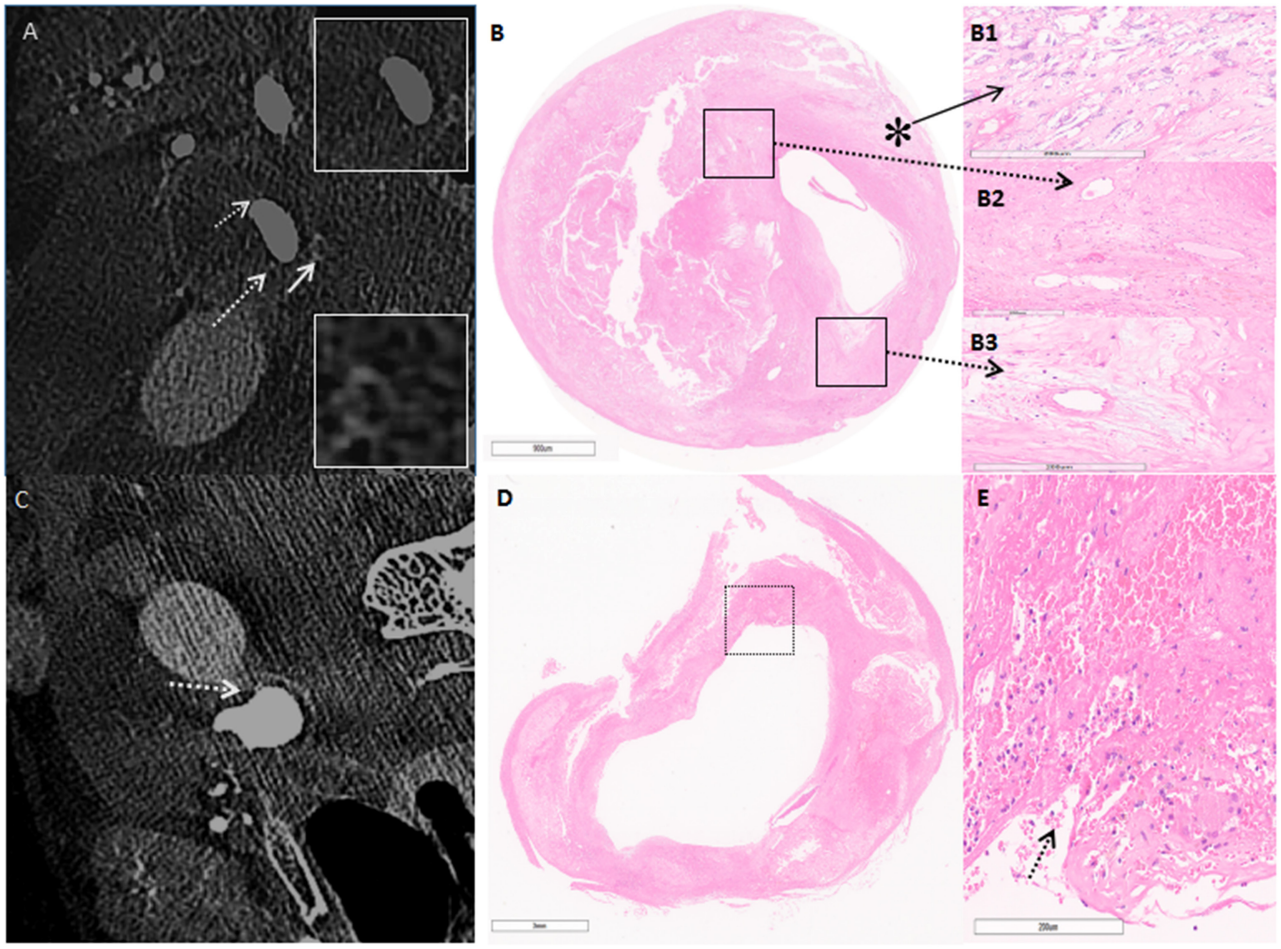
**(A)** C-arm cone-beam CT (CBCT) shows enhancement on the surface of the carotid plaque in cross-section imaging. The white arrow points to microcalcification, white broken arrow points to neovascularization. **(B)** Hematoxylin & eosin stain demonstrates the corresponding area on CBCT (5×). **(B1)** Higher power magnification shows microcalcification (asterisk) (50×). **(B2,B3)** Higher power magnification corresponding to the area outlined by the black box in **(B)**; broken arrow points to neovascularization (50×). **(C)** CBCT shows contrast enhancement from the artery lumen toward the inside of the plaque as the line shape (broken arrow). **(D)** H&E confirms fibrous rupture (5×). **(E)** Higher-power magnification corresponding to the area outlined by the black box in **(D)**; arrow points at fibrous cap rupture, and beside the rupture exists a large pool of erythrocytes (50×).

Carotid endarterectomy was performed under general anesthesia. During the operation, numerous embolic signals were detected by transcranial Doppler. C-arm CBCT showed high-density structures and linear contrast enhancement from the vascular lumen to the plaque, related to microstructure and plaque rupture. Histopathology confirmed that the high-density areas represented neovascularization and macrocalcification, with linear enhancement representing plaque rupture (Figure 2). Postoperative angiography showed that the reconstructed vascular lumen was smooth, and the arterial flow velocity had increased. MRI showed new infarcts within the basal ganglia and frontoparietal areas.

## Discussion

C-arm cone-beam CT (CBCT) has proven to be a valuable imaging technique and is increasingly used- in neuro-intervention procedures. CBCT has demonstrated effectiveness in evaluating in-stent restenosis after intracranial atherosclerosis treatment and follow-up imaging of intracranial stent therapy for aneurysms (11–13). Recently, CBCT has also been used to visualize neurovascular microanatomy of the posterior circulation. Dobrocky et al. (14) used CBCT to demonstrate the absence of pontine perforators in the fusiform vessel segment of vertebra-basilar dolichoectasia. In this case, two concentrations of diluted contrast can be used to provide the lumen and plaque characteristics to identify the detailed resolution of plaque morphology. High-resolution CBCT suggested that the VV enhancement covering the carotid plaque was closely related to unstable plaque and postoperative ipsilateral new ischemic lesions^.^ (15). As an imaging method, CBCT may be as valuable as HR-MRI in evaluating the microstructure of atherosclerosis.

VV enhancement at the level of carotid plaque on CT or MRI is associated with an increased risk of ischemic stroke (16). High-resolution CBCT can visualize the arterial wall more clearly than CT angiography, thus distinguishing proliferation of the VV covering carotid plaque and the site of microcalcification. The effect of calcification on plaque is controversial. Calcification with different amounts, size, shape, and location play inconsistent roles in plaque homeostasis (17). However, it is felt that early microcalcification reflects a vulnerable stage of plaque development. Microcalcification in the fibrous cap may increase local stress, leading to plaque instability, plaque rupture, and thrombosis (18). HR-MRI is considered excellent for evaluating plaque morphology, but it is not sensitive to the presence of microcalcification. Furthermore, different components may demonstrate similar signals, so multiple sequences are needed to determine plaque composition. In this case, HR-MRI showed vessel wall enhancement on T1 and T2 sequences. This enhancement was further confirmed by CBCT as neovascularization related to the intima. CBCT also displayed microcalcification distribution in the same layer of fibrous cap tissue and plaque rupture (Figure 2), with linear contrast enhancement from the vascular lumen to plaque tissue which corresponded to the histopathology. Although ultrasound imaging, PET-CT, and OCT can be used to identify neovascularization and microcalcification, concerns of inconsistent imaging quality, higher costs, and potential clinical risks may limit their clinical application.

Previous studies have reported that CBCT provides greater spatial resolution and discrimination in assessing both absolute luminal stenosis and plaque morphology in intracranial atherosclerotic disease (19). We demonstrate the utility of CBCT in assessing the microstructure of plaque components, including VV enhancement and plaque rupture site by histology. This is also the first report showing that microcalcifications and plaque rupture can be identified by CBCT. Thus, CBCT can be used as a promising supplement to current imaging modalities to evaluate plaque components more accurately. However, this technology needs to be further studied in the future, especially for displaying the microstructure in the early stage of atherosclerotic plaque.

## Data Availability Statement

The raw data supporting the conclusions of this article will be made available by the authors, without undue reservation.

## Author Contributions

LJ contributed to the conception and design of the case report. JD drafted the initial manuscript. XB and LX wrote sections of the manuscript. AD and TW critically reviewed and revised the manuscript. XL and YF collected the data. All authors reviewed and approved the final version of the manuscript.

## Funding

This work was supported by the Xuanwu Hospital Science Program for Fostering Young Scholars (QNPY2020003). The funders have no role in study design, data analysis, and writing for the manuscript.

## Conflict of Interest

The authors declare that the research was conducted in the absence of any commercial or financial relationships that could be construed as a potential conflict of interest. The handling editor declared a shared affiliation with several of the authors JD, XB, TW, XL, YF, and LJ at time of review.

## Publisher's Note

All claims expressed in this article are solely those of the authors and do not necessarily represent those of their affiliated organizations, or those of the publisher, the editors and the reviewers. Any product that may be evaluated in this article, or claim that may be made by its manufacturer, is not guaranteed or endorsed by the publisher.
